# MALDI Mass Spectrometry Imaging for the Simultaneous Location of Resveratrol, Pterostilbene and Viniferins on Grapevine Leaves

**DOI:** 10.3390/molecules190710587

**Published:** 2014-07-21

**Authors:** Loïc Becker, Vincent Carré, Anne Poutaraud, Didier Merdinoglu, Patrick Chaimbault

**Affiliations:** 1 Laboratoire de Chimie et Physique-Approche Multi échelle des Milieux Complexes (LCP-A2MC), Institut Jean Barriol (FR 2843), Université de Lorraine, ICPM 1 Boulevard Arago, F-57078 Metz, France; E-Mails: loic.becker@univ-lorraine.fr (L.B.); patrick.chaimbault@univ-lorraine.fr (P.C.); 2 Institut National de Recherche en Agronomie (INRA) – Santé de la Vigne et Qualité du Vin (UMR 1131), 28 rue de Herrlisheim, F-68021 Colmar, France; E-Mails: anne.poutaraud@colmar.inra.fr (A.P.); didier.merdinoglu@colmar.inra.fr (D.M.)

**Keywords:** mass spectrometry imaging, grapevine, stilbene phytoalexin, viniferin, resveratrol, pterostilbene, MALDI, *Plasmopara viticola*, *Vitis vinifera*

## Abstract

To investigate the *in-situ* response to a stress, grapevine leaves have been subjected to mass spectrometry imaging (MSI) experiments. The Matrix Assisted Laser Desorption/Ionisation (MALDI) approach using different matrices has been evaluated. Among all the tested matrices, the 2,5-dihydroxybenzoic acid (DHB) was found to be the most efficient matrix allowing a broader range of detected stilbene phytoalexins. Resveratrol, but also more toxic compounds against fungi such as pterostilbene and viniferins, were identified and mapped. Their spatial distributions on grapevine leaves irradiated by UV show their specific colocation around the veins. Moreover, MALDI MSI reveals that resveratrol (and piceids) and viniferins are not specifically located on the same area when leaves are infected by *Plasmopara viticola*. Results obtained by MALDI mass spectrometry imaging demonstrate that this technique would be essential to improve the level of knowledge concerning the role of the stilbene phytoalexins involved in a stress event.

## 1. Introduction

Mass spectrometry succeeds in providing a lot of qualitative and quantitative data on plant omics [1]. Generally, to get the information, the plant material needs to be sampled and extracted by solvents before chemical analysis. Using this approach, the sensitivity of detection is very high but the exact location of compounds of interest in tissues is lost. In just a few years, mass spectrometry imaging (MSI) made the dream of the location of molecular compounds at the micron scale to come true [2,3]. First developed on animal slices [4,5], this emerging technique finds a growing interest in plant proteomic [6] and metabolomic [7,8,9]. Indeed, MSI is able to provide a specific spatial distribution of proteins or metabolites in tissues. Depending on the ion source, the sample preparation and the mass spectrometer used, the spatial distribution of molecules on a plant sample is specifically dedicated to some compound families [10,11].

In this context, we developed a selective method to map several stilbene phytoalexins on grapevine leaves using a time-of-flight mass spectrometer (TOFMS) fitted with a laser ion source operated at 266 nm [12]. Phytoalexins are of great interest because they are biosynthetized in response to biotic or abiotic stress. They are also well known for inducing an antifungal activity [13,14,15,16,17,18] and, for example, we observed by MSI experiment a co-localization of resveratrol and pterostilbene (trimethoxystilbene) at the infection site on the leaf abaxial side (Cabernet Sauvignon) [12] after infection by downy mildew. MSI allowed the identification and mapping of resveratrol and pterostilbene but the biosynthesis of some other stilbenes is also expected to be induced in grapevineleaves and even berries in response to pathogen infection or UV irradiation [19,20,21]. Besides the glycosylation of stilbenes (e.g., piceids), oxidative oligomerization of resveratrol catalyzed by plant peroxidases may occur and several of them have been identified in stressed grapevine leaves [22,23,24]. For example, the *δ*-viniferin has been identified as the main dimer of *trans-*resveratrol synthesized in *Vitis vinifera* leaves infected by *Plasmopara viticola* [25]. These stilbene phytoalexins were found to be more or less toxic against fungi according to their chemical structure. In this field, the viniferins are known to be more active than resveratrol and thus are suspected to be highly involved against pathogen proliferation [13,26]. Resveratrol is synthesized in a large amount regardless of the cultivar, the susceptibility or the resistance to fungi. Even if resveratrol has itself an antimicrobial effect [27], its transformation to other stilbene phytoalexins could be decisive in the defense mechanisms of the grapevine. Consequently, it could be extremely informative to observe the spatial distribution of more stilbene phytoalexins than resveratrol or pterostilbene in stressed plant organs. In this paper, we also present the first MSI experiments allowing the mapping of viniferins on grapevine leaves.

In a previous paper, we reported the imaging of metabolites from *Vitis vinifera* leaves by laser desorption/ionization (LDI) time-of-flight mass spectrometry [12]. The relatively high laser power density (around 10^8^ W·cm^−1^) allowed a sufficient ion yield to highlight species of interest in the mass spectra at the grapevine leaf surface. In this configuration, we identified different molecules among which the resveratrol and the pterostilbene but also some additional compounds such as diacyl and triacyl glycerols. The gain of sensitivity to resveratrol and pterostilbene by the biphotonic ionization process at 266 nm wavelength was outstanding, but it may be counterbalanced by the signal loss of laser-sensitive substances such as viniferins. To overcome this limitation and access to the spatial distribution of other stilbene phytoalexins on plant material, we evaluate the potential of a MALDI approach which consists in the addition of a layer of an exogen compound called “matrix”, deposited on the leaf surface before the LDI process at 337 nm wavelength.

## 2. Results and Discussion

For mass spectrometry imaging purpose, MALDI has already been used to map different metabolites on plant organs [28] such as lipids on leaf surface [29], flavonoids or glycoalkaloid in roots and root nodules [30,31], sugars within seed or stem sections [32,33]. Different matrices are described in the literature for MALDI MS analysis of vegetable material such as 1,5 diamino naphthalene (DAN) or 9-amino acridine (9-AA) in negative ion mode, and tri-hydroxy acetophenone (THAP), α-cyano-4-hydroxy cinamic acid (CHCA) or 2,5 dihydroxy-benzoic acid (DHB) in positive ion mode. Graphite was also used to avoid high matrix ion background in the *m/z* range of metabolites [34] but it may lead to overmuch carbon deposit on ion lenses during experiments. Consequently, we firstly proceeded to a matrix selection for MSI of phytoalexin by analyzing the standard stilbene compounds by MALDI-TOFMS with different matrices. To induce phytoalexin synthesis in high amount, grapevine leaves were irradiated with UV-C. They were firstly extracted by methanol to control phytoalexin content using HPLC-MS/MS and MALDI-TOFMS analyses. Finally, MSI experiments were conducted on *Vitis vinefera* leaves either irradiated by UV-C or infected by *Plasmopara viticola*.

### 2.1. Stilbene Analyses by MALDI-TOFMS

#### 2.1.1. Matrix Selection for Stilbene Analyses by MALDI-TOFMS

To evaluate the most appropriate matrix for stilbene analysis, *trans*-resveratrol, pterostilbene and *trans*-δ-viniferin have been analyzed by MALDI-TOFMS in positive and negative ion mode using different matrices (Table 1). Laser desorption ionization (LDI) experiments which is our reference ionization method was carried out by depositing 1 µL of pure stilbene standard solution at 10^−5^ M on the target plate without matrix. For MALDI experiments, matrix solution at 10^−1^ M in acetonitrile/water (50/50 with or without 0.1% of TFA) was mixed with standard compound solution (matrix/analyte ratio of 1000) and deposited on the target plate (dried-droplet method). Results are given in Table 1 which represents, for each experimental condition, the signal-to-noise ratio (S/N) values of MS peaks corresponding to stilbene molecular ions.

Whether in negative or positive ion mode, resveratrol and pterostilbene are detected without any matrix. The highest sensitivity is reached for deprotonated molecular ions at *m/z* 227 and 255 respectively unlike what we observed at 266 nm [12]. As expected, *trans*-δ-viniferin is never detected in LDI conditions.

The matrix contribution to the detection of the viniferin is noteworthy. In positive ion detection, with CHCA or DHB in acid medium (TFA), the protonated molecular ion [M+H]^+^ and radical molecular ion M^●+^ of the viniferin are detected with the highest signal-to-noise ratios at *m/z* 455 and 454. With a less pronounced effect, THAP allows the radical molecular ion M^●+^ to be detected and in negative detection mode, deprotonated molecular ion is detected at *m/z* 453 by using 9-AA. Due to the matrix ion interference at *m/z* 453, DAN cannot be used as a matrix for viniferin analysis.

**Table 1:** Signal-to-noise ratio (S/N) values of Matrix Assisted Laser Desorption/Ionisation (MALDI)-time-of-flight mass spectrometer (TOFMS) peaks corresponding to stilbene molecular ions (* radical ion, otherwise, each peak corresponds to protonated ion (positive mode) or deprotonated ion (negative mode); N/A, not applicable because of the presence of an interfering peak).

Ion mode	Matrix	S/N ratio		
		*trans*-Resveratrol	Pterostilbene	*trans*-δ-Viniferin
Negative	without	307	166	/
	9-AA	68	114	11
	DAN	349	230	N/A
	CHCA	/	/	/
	THAP	/	/	/
	DHB	/	/	/
Positive	without	91	451	/
	DAN	/	/	/
	DAN+TFA	/	/	/
	DHB	25	55	139/145 *
	DHB+TFA	259	358	231/239 *
	CHCA	N/A	246	146 *
	CHCA+TFA	N/A	708	501 *
	THAP	/	/	/
	THAP+TFA	38	/	44
	9-AA	/	/	/

CHCA matrix in positive mode generates an intense mass peak at *m/z* 228 corresponding to [M+K]^+^ which interferes with the resveratrol molecular ion at the same *m/z* value. For its part, DHB matrix allows both resveratrol and pterostilbene to be detected as protonated molecular ions with a high S/N value. The addition of trifluoroacetic acid significantly improves the signal. Thus, the signal of resveratrol with DHB is only 15% lower from that obtained without matrix but it is around two times higher for pterostilbene compared to LDI. Therefore, DHB with TFA addition provides *m/z* peaks related to each tested stilbene with high sensitivity making it a matrix of choice for stilbene analysis by MALDI-TOF.

#### 2.1.2. MALDI-TOFMS Analysis of Stressed Leaf Extract

To investigate resveratrol, pterostilbene and viniferins in a real sample, a grapevine leaf has been first irradiated by UV-C. Two days after this treatment, the production of stilbene phytoalexin is expected to be induced. Stilbenes were then extracted from leaves using methanol. The leaf was then extracted with methanol. The presence of stilbenes in the leaf extract was controlled by HPLC-ESI/MS and MS/MS in negative ionization mode (more sensitive detection than in the positive mode).

**Figure 1:**
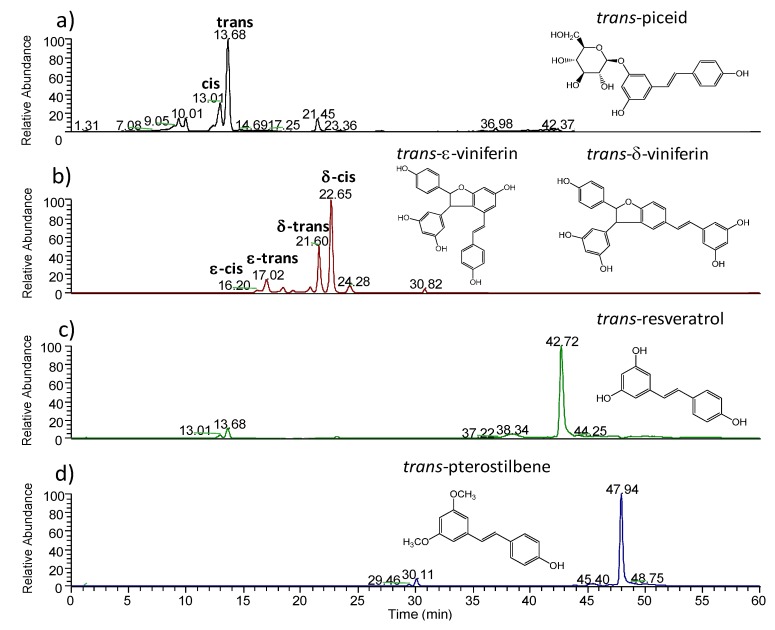
Ion extract chromatograms from HPLC-ESI/MS analysis of stressed leaf extract (**a**) piceid isomers at *m/z* 389 (**b**) viniferin isomers at *m/z* 453 (**c**) resveratrol at *m/z* 227 and (**d**) *m/z* pterostilbene at 255 – peak assignments were confirmed by MS/MS experiments of the 10 more abundant ions in each mass spectrum, the molecular structures of *trans*- isomers of each stilbene are displayed.

Several stilbenes are detected by LC-ESI/MS using a reversed-phase support (RPLC). The identification was confirmed by the retention time and the fragmentation pattern of standard compounds compared to the literature (MS/MS, Table 2) [25,35,36]. Ion extract chromatograms of deprotonated molecular ions are displayed in the Figure 1. Piceids are the most polar stilbenes due to their glucose moiety. Thus, they are the less retained stilbenes as expected in RPLC. The *trans*-isomer is eluted at 13.68 min and *cis*-isomer at 13.01 min. The peaks at 17.02 and 18.07 min are associated with the *cis*- and *trans*-ε-viniferin respectively. The following peaks at 21.60 and 22.65 min are in turn associated with *trans*- and *cis*- δ-viniferin respectively. This elution order of viniferin isomers have been described by Pezet using a C_18_ column [25]. Resveratrol is identified at 42.72 min. Finally, pterostilbene, the most apolar stilbene, is detected at 47.94 min.

**Table 2:** List of retention times, molecular formulae and MS/MS product ions of stilbenes detected by LC-ESI/MS (see also Figure 1). The relative intensities of fragments are indicated in parenthesis.

Compound	Retention Time (min)	[M-H]^−^	Precursor Ion	MS/MS (CID)
*cis*-piceid	13.01	C_16_H_15_O_3_^−^	389	228_(100)_
*trans*-piceid	13.68	C_16_H_15_O_3_^−^	389	228_(100)_
*cis*-ε-viniferin	17.02	C_28_H_21_O_6_^−^	453	435_(20)_; 411_(10)_; 369_(12)_; 359_(42)_; 347_(100)_; 333_(44)_
*trans*-ε-viniferin	18.07	C_28_H_21_O_6_^−^	453	435_(24)_; 411_(14)_; 359_(100)_; 347_(58)_; 333_(18)_
*trans*-δ-viniferin	21.60	C_28_H_21_O_6_^−^	453	435_(22)_; 411_(15)_; 359_(100)_; 347_(40)_; 333_(8)_
*cis*-δ-viniferin	22.65	C_28_H_21_O_6_^−^	453	435_(100)_; 411_(68)_; 369_(62)_; 359_(38)_; 347_(40)_ ; 333_(50)_; 317_(14)_; 307_(20)_; 267_(12)_; 251_(13)_
*trans*-resveratrol	42.72	C_14_H_11_O_3_^−^	227	185_(100)_; 183_(38)_; 159_(32)_; 157_(29)_; 143_(11)_
*trans*-pterostilbene	47.94	C_14_H_11_O_3_^-^	255	240_(100)_; 239_(5)_

**Figure 2:**
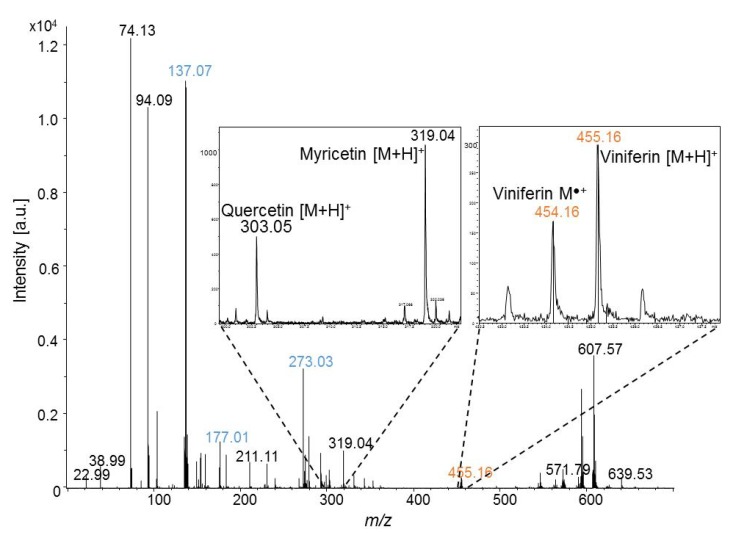
MALDI-TOF mass spectrum of methanol extract from grapevine stressed leaf – the blue peak labels correspond to matrix ions (DHB); in inserts, a zoom of *m/z* peaks corresponding to flavonol aglycone ([M+H]^+^) and viniferins (M^•+^ and [M+H]^+^).

The extraction sample of grapevine leaf irradiated by UV was also investigated by MALDI-TOFMS with DHB as the matrix (and 0.1% of TFA). The average mass spectrum obtained from 50 mass spectra is displayed in the Figure 2. In the low mass region, the mass spectrum of a methanolic extract of leaf is similar to the one obtained with pure DHB matrix with mass peaks observed at *m/z* 137, 155, 177, 193 and 273. At *m/z* higher than 500, mass peaks correspond to diacylglycerol compounds as already reported in LDI-TOFMS analysis of *Vitis vinifera* leaves. With DHB, stilbenes are detected as protonated ions. Resveratrol and pterostilbene are also detected as protonated ions but the detection of viniferin as molecular ions is worth to note. They could be observed at *m/z* 454 and 455 (right window in Figure 2). Note that viniferin isomers cannot be distinguished from each other by MS and that they all contribute to the same MS signal. Other metabolites are jointly detected as for example flavonol aglycone at *m/z* 303 for quercetin and *m/z* 319 for myricetin (left window in Figure 2). In our experimental conditions, the loss of sugar moiety systemically occurs. Thus, piceids are solely detected without sugar moiety and contribute to the same *m/z* signal as resveratrol (in the next sections, *m/z* peak at 229 will be assigned to resveratrol/piceids).

### 2.2. In-Situ MALDI-MSI of Stilbenes on Stressed Leaves

**Figure 3:**
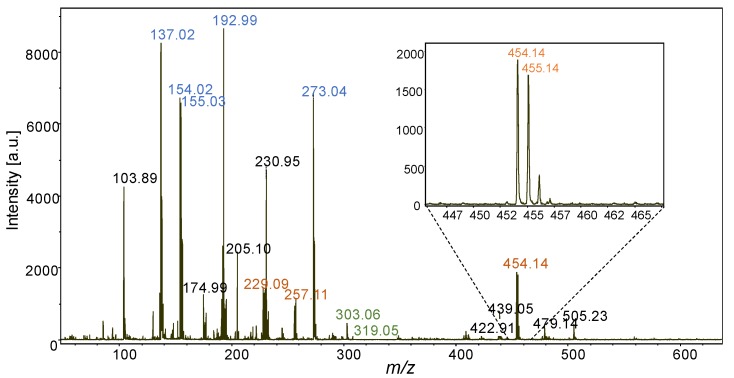
MALDI-TOF mass spectrum of stressed grapevine leaf: the blue peak labels correspond to matrix ions (DHB). Stilbene phytoalexins are detected as protonated and radical ions as it is observed for the viniferin in insert.

**Figure 4:**
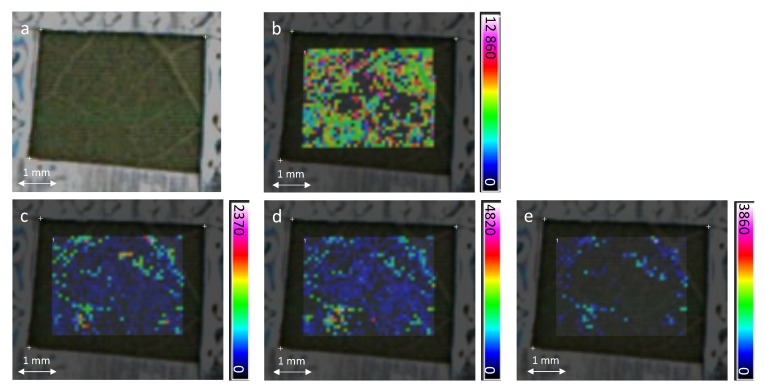
MALDI-MSI of UV stressed grapevine leaf (**a**) optical image; (**b**) ion extracted image related to one peak of the matrix [DHB-H_2_O+H]^+^ (*m/z* 137); (**c**) ion extracted image of resveratrol and piceids (*m/z* 229); (**d**) ion extracted image of pterostilbene (*m/z* 257); (**e**) ion extracted image of viniferin (*m/z* 454 and 455). The color scale indicates the absolute intensity of each pixel (arbitrary units).

Using DHB matrix for the successful detection of viniferins by MALDI-MS experiment of leaf extract is thus a promising route for the MSI investigation of stilbene compounds. 4 µL of a DHB solution were deposited with a micropipette over a 10.6 mm² area on the abaxial side of a UV-C-stressed leaf. The drying time was short enough to prevent needle formation during matrix crystallization and to avoid the distribution of the studied metabolites in the native sample to be disturbed. Moreover, the size of the image is smaller than the surface of the deposited droplet containing the matrix. Thus, the imaged area is corresponding to the center of the dried droplet where the coffee ring effect does not exist. To support this affirmation, the repartition of the ion *m/z* 137 [DHB-H_2_O]^+^ (the most intense ion of the matrix) shows a rather homogenous deposition (Figure 3). MSI experiment was then conducted on this sample. A mass spectrum corresponding to one pixel is displayed on the Figure 3. DHB mass peaks contribute to a large part of the signal (peak labels in blue). However, all previously investigated stilbenes are detected as protonated molecular ions: resveratrol/piceids at *m/z* 229, pterostilbene at *m/z* 257 and viniferins at *m/z* 455 and also radical ion at *m/z* 454 (insert of the Figure 4). Compared to MALDI-TOFMS analysis of methanolic extract, the intensity of viniferins significantly increases meaning that the MALDI-MSI improves the sensitivity of their detection. This may be explained by the fact that there is no dilution effect in MSI (metabolites are detected where they are located) whereas in MALDI-TOFMS, methanol extracts rather provide an average response of metabolites contained in the sampled leaf disc.

The spatial distributions of stilbene phytoalexins on the leaf are given in the Figure 4. The color scale represents the relative intensity of each ion. For each stilbene, the black color is used when nothing is detected in the corresponding pixel whereas the white color represents the maximum of intensity detected in the map.

Examining the ion extracted MS images of resveratrol/piceids at *m/z* 229, pterostilbene at *m/z* 257 and viniferins at *m/z* 454 and 455, the heterogeneity of their surface distributions is clearly highlighted. Moreover, they are almost exclusively localized on the same areas of the leaf. Their spatial location evidences a clear relationship in their synthesis under UV-stress conditions. Even if the image resolution is not sufficient to explore cells at the organelle scale, stilbenes seem to accumulate themselves in the network of small veins and more precisely in the dense parenchyma tissue. This is in good agreement with UV irradiated leaf analyzed by fluorescence for which global stilbene fluorescence has been mainly located in vein and lignified tissues [37,38]. Notably, none of these stilbenes was detected in the control samples (not irradiated) leading to black images (data not shown).

**Figure 5:**
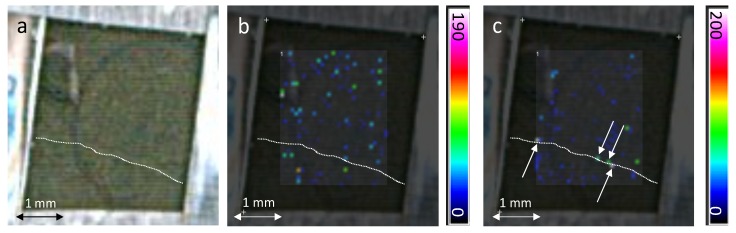
MALDI-MSI of grapevine leaf 5 days after infection by *Plasmopara viticola* (**a**) optical image; (**b**) ion extracted image of resveratrol/piceids at *m/z* 229; (**c**) ion extracted image of viniferins at *m/z* 454 and 455. The color scale indicates the absolute intensity of each pixel (arbitrary units). The dotted line highlights a small vein and the arrows point out pixels corresponding to a high intensity of viniferins.

The MSI experiment was then conducted on Cabernet Sauvignon leaf infected by *Plasmopara viticola* (Figure 5). Before analysis, 4 µL of DHB matrix solution were deposited with a micropipette over a 9.1 mm² area on the grapevine leaf five days after infection. The MS image of the control leaves did not exhibit any stilbene (data not shown) because their concentration is under the detection limits. The Figure 4 displays ion extraction images of resveratrol/piceids and viniferins. As it was observed under a UV stress, their spatial distribution on the grapevine leaf is non-homogeneous. However, some differences appear between resveratrol/piceids and viniferin localizations (for this sample, the pterostilbene level was under the detection limit). While viniferins are mainly located around the veins, the distribution of resveratrol/piceids is more scattered on the leaf surface. Its distribution should correspond to the infection sites. Grapevine reacts to *P. viticola* infection by producing high amounts of stilbenes at the infection site [15] and MALDI-MSI brings new chemical details on their spatial distributions. Resveratrol and piceid are much less toxic against *P. viticola* than viniferins [14]. Viniferins are supposed to be involved depending on the cultivar resistance to the pathogen [22]. For the Cabernet Sauvignon, which has a low degree of resistance to *P. viticola*, the less toxic compounds for pathogen are localized at the leaf infection sites where the viniferins are not accumulated. It suggests that for a susceptible variety, the viniferins may be too far away from infection sites to play a real antifungal role. Their specific locations need to be understood according to the involved biosynthesize pathways and their isomeric composition [21]. Consequently, the investigation of viniferins locations from a range of susceptible to resistant grapevine species is now possible by MALDI-MSI to understand their role in the constitutive and inducible defenses of grapevine against fungi.

## 3. Experimental Section

### 3.1. Plant Material and Leaf Sample Preparation

The study was carried out on Cabernet Sauvignon, a grape variety of *Vitis vinifera* highly sensitive to *Plasmopara viticola*. Plants were grown in greenhouse. The sixth leaf, counted from the apex of 3.5 months old plants having 12–14 fully expanded leaves, was harvested and washed with demineralized water. To induce stilbene synthesis, grapevine leaves were irradiated by UV-C lamp at the 254 nm wavelength (Osram, 30 W, 90 µW·cm^−2^, Molsheim, France) for 180 s or were infected by spraying a 2 × 10^4^ sporangia·mL^−1^ solution of *Plasmopara viticola* sporangia on the abaxial side. After inoculation or irradiation, leaves were transferred to wet paper with the abaxial surface up in trays closed by transparent plastic bags. Leaves were stored in a culture chamber for an initial period at a temperature of 23 °C in the dark for 24 h, then 18 h of light (about 200 µmol·m^−2^·s^−1^) and 6 h of darkness until analysis. 

Two days after irradiation or five days after infection, foliar discs were cut out using a 2 cm-diameter hollow-punch. Leaf discs were placed in a high vacuum to stop phytoalexin synthesis and stored at 4 °C before imaging experiments. For MALDI experiments, matrix solution at 10^−1^ mol∙L^−1^ in a solvent mix (acetonitrile/water—50/50) acidified by TFA was deposited on the leaf sample by using a P20 micropipette (Eppendorf).

### 3.2. Leaf Extraction

Solid-liquid extraction of the leaf was performed as follows: leaf disc placed in 0.5 mL of methanol and heated at 60 °C for 45 min under stirring. Then, the leaf disc was removed from the extract which was centrifuged at 12,000 rpm before MS analysis.

### 3.3. Standard and Solvents

Pure *trans*-resveratrol and *trans*-pterostilbene were purchased from Sigma-Aldrich. Viniferins were collected from semi-preparative LC of methanol extraction of stressed *Vitis* leaves. All matrices, 9-amino-acridine (9-AA), 1,5-diaminonaphtalene (DAN), 2,5-dihydroxybenzoic acid (DHB), α-cyano-4-hydroxycinnamic acid (CHCA) and trihydroxyacetophenone (THAP) were purchased from Sigma-Aldrich. Trifluoroacetic acid (TFA) was purchased from Sigma-Aldrich. HPLC-grade methanol, acetonitrile and water were purchased from VWR. ESI positive and negative calibration kit from Thermo was used to achieve mass calibration of ESI-LTQ system.

### 3.4. LC-MS and MS/MS

For LC-MS analysis, high performance liquid chromatography system (Dionex Ultimate 3000, Dionex, France) was connected to a dual-pressure linear ion trap mass spectrometer (LTQ Velos Pro, Thermo Fisher Scientific, San José, CA, USA). For stilbene separation, C18 reverse phase column was used (Symmetry Shield, 4.6 × 50 mm, 3.5 µm, Waters). 20 µL of sample were injected. The flow rate was kept to 500 µL·min^−1^ and a constant elution gradient was applied from 0 (5% acetonitrile/95% water) to 55 min (100% acetonitrile) during the LC run. HESI (*Heated Electrospray Ionization Source*) interface was plugged to the ion source of the LTQ mass spectrometer. MS system was running from 110 to 2000 *m/z* at MS scan rate of 9 Hz. To confirm chromatographic peak assignment, MS/MS by CID was systematically conducted on the most intense 10 mass peaks of each mass spectrum.

### 3.5. MALDI Mass Spectrometer

A Bruker Reflex IV MALDI–TOF mass spectrometer (Bruker Daltonics, Bremen, Germany) was used to perform *in situ* MALDI analysis and imaging experiments. The nitrogen laser generates a laser pulse at a wavelength of 337 nm with a pulse duration of 4 ns and a 9 Hz repetition rate (Science Inc., Boston, MA, USA). Positive mass spectra were acquired in the 0–1000 *m/z* range. The mass spectrometer was operated in the reflectron mode at a total acceleration voltage of 20 kV and a reflecting voltage of 23 kV. A delay time of 200 ns was used prior ion extraction. The used laser fluence was kept at ~0.5 J/cm^2^.

### 3.6. Mass Spectrometry Imaging (MSI)

For MSI experiments, leaf discs were fixed on a metal MALDI target plate with aluminized tape. FlexImaging software (Bruker) allowed the tracking of the leaf sample on MALDI target plate, the image pixel features and the treatment of post-acquisition image to be achieved. The mass spectrum obtained for each pixel of the images corresponds to the averaged mass spectrum of 50 consecutive mass spectra on the same location. Approximately 3 h were required to achieve an image of 7.5 mm² area with a 75 µm spatial resolution (1100 pixels)*.*

## 4. Conclusions

For the first time, MALDI was successfully conducted on stilbene phytoalexins and more particularly on the viniferins. From all tested matrices, the DHB allows the viniferins, resveratrol and pterostilbene to be detected with a higher sensitivity. The *in-situ* grapevine leaf response to a stress was then investigated using MALDI imaging mass spectrometry experiment. For this purpose, stress was generated by UV-C irradiation. The ion images of resveratrol/piceids, pterostilbene and viniferins exhibit heterogeneous distribution on leaf surface but also demonstrate their colocalization around the leaf veins. Moreover, the MALDI-MSI investigation of a Cabernet Sauvignon leaf infected by *Plasmopara viticola* allows different spatial distributions between resveratrol/piceids and viniferins to be highlighted. Only resveratrol/piceids, the less toxic compounds for fungi are detected on the infection sites of this susceptible cultivar. This result suggests that viniferin locations may influence the resistance level to a pathogen. The MALDI mass spectrometry imaging of stilbene phytoalexins provides a new level of understanding the plant response to a biotic or an abiotic stress.
